# The prognostic impact of left ventricular thrombus resolution after acute coronary syndrome and risk modulation via antithrombotic treatment strategies

**DOI:** 10.1002/clc.23741

**Published:** 2021-10-19

**Authors:** Felix Hofer, Niema Kazem, Ronny Schweitzer, Patricia Horvat, Max‐Paul Winter, Lorenz Koller, Christian Hengstenberg, Patrick Sulzgruber, Alexander Niessner

**Affiliations:** ^1^ Division of Cardiology, Department of Internal Medicine II Medical University of Vienna Vienna Austria

**Keywords:** acute coronary syndrome, antithrombotic therapy, left ventricular thrombus

## Abstract

**Background:**

Left ventricular thrombus (LVT) is a rare but dreaded complication during the acute phase of acute coronary syndrome (ACS). However, profound data on long‐term outcome and associated antithrombotic treatment strategies of this highly vulnerable patient population are scarce in current literature.

**Methods:**

Patients presenting with ACS were screened for presence of LVT and subsequently included within a prospective clinical registry. All‐cause mortality and the composite of major adverse cardiac events (MACE) and thromboembolic events were defined as primary and secondary endpoint.

**Results:**

Within 43 patients presenting with LVT, thrombus resolution during patient follow‐up was observed in 27 individuals (62.8%). Patients that reached a resolution of LVT experienced lower incidence rates of death (−23.9%; *p* = .022), MACE (−37.8%; *p* = .005), and thromboembolic events (−35.2%; *p* = .008). Even after adjustment for clinical variables, thrombus resolution showed an independent inverse association with all‐cause death with a hazard ratio (HR) of 0.14 (95% CI: 0.03–0.75; *p* = .021) and as well with MACE with a HR of 0.22 (95% CI: 0.07–0.68; *p* = .008) and thromboembolic events with a HR of 0.22 (95% CI: 0.06–0.75; *p* = .015). Triple antithrombotic therapy (TAT) with ticagrelor/prasugrel showed a strong and independent association with thrombus resolution with an adjusted HR of 3.25 (95% CI: 1.22–8.68; *p* = .019) compared to other strategies.

**Conclusion:**

The presented data indicate a poor outcome of ACS patients experiencing LVT. In terms of a personalized risk stratification, thrombus resolution has a strong protective impact on both all‐cause death and MACE with the potential to tailor treatment decisions—including an intensified antithrombotic treatment approach—in this patient population.

**Abbreviations**
ACS: acute coronary syndrome
CI: confidence interval
CK: creatine kinase
CMR: cardiac magnetic resonance tomography
DAPT: dual antiplatelet therapy
DAT: dual antithrombotic therapy
HR: hazard ratio
IQR: interquartile range
LAT: left atrial thrombus
LVEF: left ventricular ejection fraction
LVT: left ventricular thrombus
MACE: major adverse cardiac events
MI: myocardial infarction
NOACS: non‐vitamin‐K antagonists
NSTEMI: non‐ST‐elevation myocardial infarction
OAC: oral anticoagulation
STEMI: ST‐elevation myocardial infarction
TAT: triple antithrombotic therapy
TTE: transthoracic echocardiography
VKA: vitamin K antagonists

## 1 INTRODUCTION

Left ventricular thrombus (LVT) is a rare complication after acute coronary syndrome (ACS), especially occurring in patients presenting late with ST‐elevation myocardial infarction (STEMI).^1^ Incidence rates differ among the observational studies from 1.6% up to 39% indicating that many LVT cases might remain undetected.^2^, ^3^, ^4^, ^5^, ^6^, ^7^ This substantial variation in the incidence rate, is caused by varieties in the imaging modality used for diagnosis and the timing and frequency of screening. Additionally, the use of modern revascularization therapies has reduced the occurrence of LV‐thrombus formation.^2^, ^3^, ^4^, ^5^, ^6^, ^7^ While the prognosis of patients presenting with LVT after ACS has been controversially discussed, it seems intuitive that individuals without thrombus resolution have an increased risk for cardiovascular events and mortality. Thrombus formation is significantly associated with anterior myocardial infarction (MI) and confers an increased risk for thromboembolic events (mostly cerebrovascular).^8^, ^9^, ^10^ In the pre‐thrombolytic era, these complications were described in approximately 10% of cases, whereas in the era of thrombolytic therapy embolic events occurred in 2%–3% of cases.^8^, ^9^, ^10^ Until now there is scarce evidence regarding the incidence of embolic events in patients treated by primary percutaneous coronary intervention (PCI) that receive dual antiplatelet therapy (DAPT) or even triple antithrombotic therapy (TAT; oral anticoagulation [OAC] plus DAPT) or dual antithrombotic therapy (DAT) as the combination of OAC with only one antiplatelet agent. However, optimal pharmacological therapy—used to reduce complications of LVT—remains challenging. While patients post MI requires DAPT for reduction of atherothrombotic risk, they also need OAC in case of LVT formation for reduction of related complications, with subsequent high risk of bleeding.^11^ For patients with large anterior STEMI, DAPT with a potent P2Y_12_ inhibitor may even be an attractive option, as potent DAPT has been shown to contribute to a lower incidence of LVT.^12^

Considering a strong impact of LVT on patient outcome and the notion that many LVTs remain undetected in clinical practice, patient characteristics that help to identify ACS individuals at risk for the development of LVT with an adverse outcome should be considered in terms of a personalized secondary prevention. However, profound data on long‐term outcome of this highly vulnerable patient population are scarce in current literature. Therefore, we aimed to investigate the impact of LVT resolution and associated antithrombotic treatment strategies on patient's outcome from a long‐term perspective.

## 2 METHODS

### 2.1 Study population and patient selection

Patients presenting with ACS (n = 2011) who underwent treatment at the Vienna General Hospital, a university affiliated tertiary care center with a high‐volume cardiac catheterization unit in the time period between January 2015 and September 2019 were screened for presence of LVT. Out of the source population a total of 52 patients (2.6%) presented with LVT after MI. Six individuals died before hospital discharge and three did not receive follow‐up imaging and were subsequently excluded for the final analysis—resulting in a total study population of 43 patients for the present long‐term analysis. All patients were older than 18 years. The study was conducted in accordance to the current criteria of the Declaration of Helsinki and was approved by the ethics committee of the Medical University of Vienna (1702/2019). Based on the study design the investigation was conducted without informed consent due to the minimal risk of study inclusion. After completion of follow‐up, the study population was stratified in patients with thrombus resolution and individuals without thrombus resolution.

### 2.2 Data acquisition and patient follow‐up

Patient‐relevant characteristics were assessed via the patients' electronic medical records of the Vienna General Hospital, as well during a standardized follow‐up procedure. Data assessment was performed by specially trained chart reviewers that inserted predefined patient characteristics into a record abstraction form for further analysis of the registry at the time of hospitalization and re‐evaluation during the entire hospitalization. Discharge letters of all participants were screened for the antithrombotic treatment approach at the time of discharge.

Patients were invited to the local department for screening of thrombus resolution. The presence of LVT was validated by contrast transthoracic echocardiography (TTE) and/or cardiac magnetic resonance tomography (CMR) of all enrolled individuals. Clinically relevant data, including the antithrombotic treatment and adherence to medication was assessed during the follow‐up visit. Thrombus resolution was defined as lack of evidence of thrombus mass in follow‐up contrast TTE or CMR.

### 2.3 Endpoint definition

All‐cause death (including death from cardiovascular, renal, and cancer disease) was chosen as primary study endpoint. The composite of major adverse cardiac events (MACE), defined as a nonfatal MI, nonfatal stroke, and cardiovascular death as well as thromboembolic events were chosen as secondary endpoints and assessed during follow‐up. The patients' cause and date of death was assessed by screening the national registry of death until December 2019 via the Austrian Registry of Death (Statistics Austria, Vienna, Austria). Causes of death were defined according to the International Statistical Classification of Disease and Related Health Problems 10th Revision.

### 2.4 Statistical analysis

Continuous data are presented as median and the respective interquartile range and analyzed using Mann Whitney U test. Categorical parameters are presented as counts and percentages and analyzed using Chi‐square test. Univariate and multivariate Cox proportional hazard models were applied to assess the influence of thrombus resolution on primary and secondary endpoints and to assess the impact of a TAT with newer P2Y_12_ antagonists on LVT resolution. Results were presented as hazard ratio (HR) and the respective 95% confidence interval (CI). A three‐step adjustment approach was followed within the multivariate regression model including comprehensive adjustment for patient characteristics (= Model 1: age and sex), clinical presentation (= Model 2: STEMI and heart failure), and laboratory values (= Model 3: NT‐proBNP and creatine kinase [CK]).

Continuous variables were log‐transformed prior to inclusion in the regression analysis. Kaplan Maier charts were plotted to graphically illustrate the impact of LVT resolution on all‐cause death, MACE and thromboembolic events and compared using log‐rank test. Statistical significance was defined by two‐sided *p*‐values <.05. Statistical analyses were performed using SPSS 26.0 (IBM SPSS, NY, USA).

## 3 RESULTS

Detailed baseline characteristics for the study population presenting with LVT (n = 43), stratified in individuals with thrombus resolution and without thrombus resolution are summarized in Table 1.

**TABLE 1:** Baseline characteristics

	Overall (n = 43)	No resolution (n = 16)	Resolution (n = 27)	*p*‐value
Clinical presentation				
Age, years (IQR)	63 (58–69)	68 (61–72)	62 (56–67)	.074
Gender (male), n (%)	38 (88.4)	14 (87.5)	24 (88.9)	.892
BMI, kg/m^2^ (IQR)	26.2 (24.2–29.5)	26.1 (24.1–29.2)	26.3 (24.2–30.2)	.756
PCI, n (%)	33 (76.7)	10 (62.5)	23 (85.2)	.093
STEMI, n (%)	26 (60.5)	8 (50.0)	18 (66.7)	.289
Anterior wall infarction, n (%)	43 (100)	16 (100)	27 (100)	1.000
*Systolic BP, mmHg* (IQR)	135.0 (115.8‐149.3)	135.5 (109.8–148.3)	135.0 (116.0–150.0)	.836
*Diastolic BP, mmHg* (IQR)	74.0 (68.0–90.0)	74.0 (66.5–89.0)	74.0 (69.5–90.0)	.785
LVEF <50% (%)	21 (48.8)	7 (43.8)	14 (51.9)	.612
Comorbidities				
Diabetes, n (%)	7 (16.3)	2 (12.5)	5 (18.5)	.610
Hyperlipidemia, n (%)	21 (48.8)	6 (37.5)	15 (55.6)	.258
Hypertension, n (%)	31 (72.1)	12 (75.0)	19 (70.4)	.746
Nicotine, n (%)	22 (51.2)	6 (37.5)	16 (59.3)	.173
Prior MI. n (%)	15 (34.9)	5 (31.3)	10 (37.0)	.704
Medication				
Beta blockers, n (%)	41 (95.3)	16 (100)	25 (92.6)	.271
ACEI, n (%)	31 (72.1)	12 (75.0)	19 (70.4)	.746
ATI, n (%)	8 (18.6)	1 (6.3)	7 (25.9)	.113
Antithrombotic treatment approach				
Acetylsalicylic acid, n (%)	43 (100)	16 (100)	27 (100)	1.000
Clopidogrel, n (%)	22 (51.2)	9 (56.3)	13 (48.1)	.612
Ticagrelor/prasugrel, n (%)	8 (7.0)	0 (−)	8 (29.6)	***.016***
DAT, n (%)	13 (30.2)	7 (43.8)	6 (22.2)	.137
TAT, n (%)	30 (69.8)	9 (56.3)	21 (77.8)	.137
TAT with clopidogrel, n(%)	22 (73.3)	9 (100)	13 (61.9)	***.031***
Duration of clopidogrel (months), median (IQR)	12 (12–12)	12 (12–12)	12 (12–12)	1.000
TAT with ticagrelor/prasugrel, n (%)	8 (26.7)	0 (−)	8 (38.1)	***.031***
Duration of ticagrelor/prasugrel (months), median (IQR)	12 (12–12)	–	12 (12–12)	NA
VKA, n (%)	33 (76.7)	13 (81.3)	20 (74.1)	.595
NOAC, n (%)	10 (23.3)	3 (18.8)	7 (25.9)	.595
Duration of OAK (months), median (IQR)	24 (12–72)	24 (18–64)	12 (12–84)	.780
Laboratory variables				
NTproBNP, pg/ml (IQR)	1526.0 (609.3–7012.3)	2945.0 (1040.3–20355.0)	1308.0 (401.8–4284.8)	.089
Troponin T max., μg/ml median (IQR)	2339.5 (224.5–5828.0)	1324.5 (44.5–3583.0)	3502.0 (548.8–7455.5)	.431
CK, U/I, median (IQR)	986.0 (119.5–2906.5)	222.0 (68.0–1545.0)	1355.0 (281.5–3431.3)	.066
CK‐MB, U/I median (IQR)	191.5 (49.0–283.0)	77.0 (26.0–465.0)	200 (61.0–296.0)	.455

*Note*: Categorical data are presented as counts and percentages and analyzed using Chi‐square‐test. Continuous data are presented as median and the respective interquartile range and analyzed using Mann Whitney U test.

Abbreviations: ACEI, angiotensin converting enzyme inhibitor; AF, atrial fibrillation; ATI, angiotensin II receptor inhibitor; BP, blood pressure; BMI, body‐mass index; CK, creatinine kinase; DAPT, dual antiplatelet therapy; DAT, dual antithrombotic therapy; INR, international normalized ratio; IQR, interquartile range; LVEF, left ventricular ejection fraction; MI, myocardial infarction; NOAC, non‐vitamin‐K anticoagulant; NT‐proBNP, N‐terminal pro b‐type natriuretic peptide; PCI, percutaneous coronary intervention; STEMI, ST‐elevation myocardial infarction; TAT, triple antithrombotic therapy; VKA, vitamin‐K antagonist.

In short, the present study population (median age: 63 years [IQR 58–69]; 88.4% male gender) covered a representative number of participants presenting with STEMI (n = 26; 60.5%) and all patients developed LVT after anterior wall infarction. The remaining 39.5% of patients presented with non‐ST‐elevation myocardial infarction (NSTEMI). In 97.7% of cases, LVT was diagnosed via TTE and in 2.3% via CMR. The median time of thrombus detection after the acute event was 5 days (IQR 3–15). The median time between thrombus detection and the first follow‐up imaging was 14 weeks (IQR 6–22).

Comparing characteristics of patients with thrombus resolution and without thrombus resolution, we observed balanced frequencies of cardiovascular risk factors, such as diabetes mellitus (*p* = .610), hyperlipidemia (*p* = .258) and hypertension (*p* = .746). Established risk factors for the development of LVT—such as reduced left ventricular ejection fraction (LVEF) (48.8%), anterior wall infarction (100%), and elevated NT‐proBNP values (median: 1526.0 [IQR 609.3–7012.3]) reflecting cardiac strain were observed with high frequencies within the study population. However, we did not observe any significant group differences. Detailed baseline echocardiographic parameters are shown in Table 2. In short, severe left ventricular dysfunction was common (median LVEF 36.0% [IQR 33.0–45.0]) with no differences between both groups (*p* = .908). Left ventricular aneurysms were found in 27.9% of patients (n = 12) without any group differences (*p* = .286). Median time of thrombus resolution was 14 weeks (IQR 6–23).

**TABLE 2:** Baseline echocardiographic parameters

	Overall (n = 43)	No resolution (n = 16)	Resolution (n = 27)	*p*‐value
Left ventricular ejection fraction, %	36.0 (33.0–45.0)	39.0 (22.0–50.0)	36.0 (35.0–40.0)	.908
End‐diastolic left ventricular diameter, mm	49.0 (43.0–55.0)	49.0 (43.0–57.0)	48.0 (42.8–53.3)	.586
End‐diastolic right ventricular diameter, mm	33.0 (29.0–36.0)	35.0 (29.5–40.0)	31.0 (28.0–34.0)	.059
Interventricular septum thickness, mm	13.0 (11.5–14.3)	13.0 (11.3–14.0)	13.0 (11.5–14.5)	.617
Left ventricular thrombus				
Area, cm^2^	2.1 (0.9–4.2)	3.1 (0.8–5.0)	2.1 (1.6–4.1)	.956
Volume, cm^3^	1.7 (1.1–4.2)	2.1 (0.4–4.8)	1.7 (1.1–4.2)	.977
Apical thrombus, n (%)	43 (100)	16 (100)	27 (100)	1.000
Left ventricular aneurysm, n (%)	12 (27.9)	6 (37.5)	6 (22.2)	.286

*Note*: Categorical data are presented as counts and percentages and analyzed using Chi‐square‐test. Continuous data are presented as median and the respective interquartile range and analyzed using Mann Whitney U test.

### 3.1 Follow‐up and outcome analysis

After a median follow‐up time of 108 weeks (IQR 68–173), 16.3% of patients died (n = 7), with 31.3% of individuals (n = 5) in the no LVT resolution subgroup and 7.4% (n = 2) in the LVT resolution subgroup, respectively (*p =* .022) (Table 3). Cardiovascular death occurred in 9.3% of patients with LVT (n = 4) with a non‐significant lower event rate in the LVT resolution group (3.7% vs. 18.8%; *p =* .062). In total, MACE occurred in 32.6% (n = 14) of cases with a LVT, resulting in a significantly lower rate of MACE in the resolution group compared to the no resolution group (18.5% vs. 56.3%; *p* = .005). Thromboembolic events occurred in 27.9% of cases (n = 12), including 14.8% (n = 4) in the LVT resolution subgroup and 50.0% (n = 8) in the no LVT resolution subgroup (*p* = .008). Major bleeding events occurred in 9.3% of individuals (n = 4) presenting with LVT, without significant subgroup differences (*p* = .296). (Table 3) LVT resolution proved to be inversely associated with long‐term mortality, presenting with a crude HR of 0.18 (95% CI: 0.03–0.93; *p* = .041). Three different multivariate models (1. patient characteristics, 2. clinical presentation, and 3. laboratory values) were used to analyze whether the prognostic value of LVT was independently associated with mortality, MACE, and thromboembolic events (Table 4). Within the multivariate model 2, LVT resolution remained inversely associated with long‐term mortality with an adjusted HR of 0.14 (95% CI: 0.03–0.75; *p* = .021) (Table 4).

**TABLE 3:** All outcomes

	Overall (n = 43)	No resolution (n = 16)	Resolution (n = 27)	Log rank test *p*‐value
MACE, n (%)	14 (32.6)	9 (56.3)	5 (18.5)	**.005**
CV death, n (%)	4 (9.3)	3 (18.8)	1 (3.7)	.062
All‐cause death, n (%)	7 (16.3)	5 (31.3)	2 (7.4)	**.022**
Thromboembolic events, n (%)	12 (27.9)	8 (50.0)	4 (14.8)	**.008**
Major bleeding (BARC 2/3), n (%)	4 (9.3)	2 (12.5)	2 (7.4)	.296

*Note*: Categorical data are presented as counts and percentages and analyzed using Log rank test.

Abbreviations: CV death, cardiovascular death; MACE, major adverse cardiac events.

**TABLE 4:** Unadjusted and adjusted effects of LVT resolution on long‐term mortality, MACE and thromboembolic events

	All‐cause death		MACE		Thromboembolic events	
	HR (95% CI)	*p*‐value	HR (95% CI)	*p*‐value	HR (95% CI)	*p*‐value
Univariate	0.18 (0.03–0.93)	**.041**	0.26 (0.09–0.77)	**.015**	0.23 (0.07–0.78)	**.018**
Multivariate						
Model 1^a^	0.22 (0.04–1.21)	.081	0.24 (0.08–0.71)	**.010**	0.21 (0.06–0.72)	**.013**
Model 2^b^	0.14 (0.03–0.75)	**.021**	0.22 (0.07–0.68)	**.008**	0.22 (0.06–0.75)	**.015**
Model 3^c^	1.10 (0.12–9.73)	.932	0.31 (0.09–1.03)	.052	0.24 (0.07–0.86)	**.029**

*Note*: Univariate and multivariate Cox proportional hazard models were applied to assess the effect of LVT resolution on all‐cause death, MACE and thromboembolic events. The *p* values in bold indicate a value of <.05.

Abbreviations: CI, confidence interval; HR, hazard ratio.

^a^ Model 1 was adjusted for age and sex.

^b^ Model 2 was adjusted for STEMI and heart failure.

^c^ Model 3 was adjusted for NT‐proBNP and CK values.

In addition, LVT resolution was also associated with a significant lower risk of MACE with a crude HR of 0.26 (95% CI: 0.09–0.77; *p* = .015) and thromboembolic events with a crude HR of 0.23 (95% CI: 0.07–0.78; *p* = .018). LVT resolution remained inversely associated with MACE, after adjustment for model 1 (adj. HR of 0.24 [95% CI: 0.08–0.71]; *p* = .010) and model 2 (adj. HR of 0.22 [95% CI: 0.07–0.68]; *p* = .008) and thromboembolic events after adjustment for model 1 (adj. HR of 0.21 [95% CI: 0.06–0.72]; *p* = .013), model 2 (adj. HR of 0.22 [95% CI: 0.06–0.75]; *p* = .015) and model 3 (adj. HR of 0.24 [95% CI: 0.07–0.86]; *p* = .029). (Table 4).

Event rates for all‐cause death, MACE, and thromboembolic events at 1 year were 3.7%, 14.8%, and 11.1% in the thrombus resolution group, compared to 25.0%, 43.8%, and 43.8% in the no thrombus resolution group, respectively.

**FIGURE 1:**
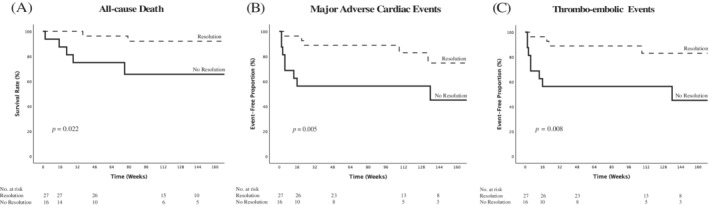
Survival curves of (A) all‐cause death, (B) MACE, and (C) thromboembolic events. Comparison of survival, MACE, and thromboembolic events between patients with left ventricular thrombus resolution and without left ventricular thrombus resolution. MACE, major adverse cardiac events

The Kaplan Meier survival plot and log‐rank test indicated a higher risk of long‐term death (*p* = .022), MACE (*p* = .009) and thromboembolic events (*p* = .010) for individuals without LVT resolution as compared to patients with LVT resolution. (see Figure 1).

### 3.2 Antithrombotic treatment strategies

Considering antithrombotic treatment strategies, we observed that all patients received OAC including 10 patients (23.3%) receiving non‐vitamin‐K oral anticoagulants (NOACs) and 33 patients (76.7%) vitamin K antagonists (VKA) respectively immediately after diagnosis. Median duration of anticoagulation therapy was 24 weeks (12–72) without significant difference between both groups. The fraction of individuals receiving DAT or TAT did also not differ significantly between both groups (*p* = .137). However, the use of TAT with a more potent P2Y_12_ inhibitor such as ticagrelor or prasugrel was observed only in the thrombus resolution group (38.1% vs. 0%; *p* = .031). Median duration of antiplatelet therapy with ticagrelor/prasugrel was 12 months, with a similar duration of 12 months for antiplatelet therapy with clopidogrel—there were no significant differences between both groups. Most importantly TAT with either ticagrelor or prasugrel showed a strong and independent association with thrombus resolution with a crude HR of 3.67 (95% CI: 1.53–8.81; *p* = .004). Notably, the prognostic impact remained stable after adjustment for model 1 (adj. HR of 3.69 [95% CI: 1.53–8.91; *p* = .004]), model 2 (adj. HR of 3.25 [95% CI: 1.22–8.68; *p* = .019]), and model 3 (adj. HR of 2.69 [95% CI: 1.10–6.58; *p* = .030]). (Table 5).

**TABLE 5:** Unadjusted and adjusted effects of TAT with ticagrelor/prasugrel on thrombus resolution

	HR (95% CI)	*p*‐value
Univariate	3.67 (1.53–8.81)	**.004**
Multivariate		
Model 1^a^	3.69 (1.53–8.91)	**.004**
Model 2^b^	3.25 (1.22–8.68)	**.019**
Model 3^c^	2.69 (1.10–6.58)	**.030**

*Note*: TAT with ticagrelor/prasugrel. Univariate and multivariate Cox proportional hazard models were applied to assess the effect of TAT with ticagrelor/prasugrel on LVT resolution. The *p* values in bold indicate a value of <.05.

Abbreviations: CI, confidence interval; HR, hazard ratio.

^a^ Model 1 was adjusted for age and sex.

^b^ Model 2 was adjusted for STEMI and heart failure.

^c^ Model 3 was adjusted for NT‐proBNP and CK values.

## 4 DISCUSSION

The current analysis is—to the best of our knowledge—one of the first in literature that investigated the impact of LVT resolution after ACS on cardiovascular events and mortality. The present data illustrates that LVT resolution was independently associated with a favorable long‐term outcome and survival free of MACE and thromboembolic events. In addition, our data indicates that TAT with potent P2Y_12_ inhibitor might be considered in patients with LVT.

Within the present investigation, we observed an incidence rate of LVT after ACS of 2.5%. Reported incidence rates vary from 1.6% up to 39%.^2^, ^3^, ^4^, ^5^, ^6^, ^7^ The principal cause of these variations is rooted in the use of modern revascularization therapies^2^, ^3^, ^4^, ^5^, ^6^, ^7^ Furthermore, different imaging modalities and the timing and frequency of screening may affect the incidence rate.^2^, ^3^, ^4^, ^5^, ^6^, ^7^

Within our study, we observed a high prevalence of established risk factors for LVT as well as high values of NT‐proBNP, Troponin T, and CK indicating extensive tissue damage and scar formation. All patients developed LVT after anterior wall infarction, which is in line with previous studies showing a significant association between LVT formation and left anterior descending artery as the culprit lesion.^7^ Compared with the existing literature, the number of patients with NSTEMI in our study is high. However, because the proportion of NSTEMI patients compared to STEMI patients is increasing significantly in clinical practice, we assume more NSTEMI patients in total.

Optimal pharmacological therapy in this highly vulnerable patient population—used to reduce complications of LVT—remains challenging. Within the present investigation VKA is the primary anticoagulant used with low prescription rates of NOACs, which is probably caused by the lack of evidence in the treatment of LVT.^11^ Furthermore, we observed that one third of patients did not achieve LVT resolution, despite additional anticoagulation. Our data indicates that the current antithrombotic strategy needs to be improved to reduce associated clinical complications. In line with our findings, a recently published study by Lattuca and colleagues observed thrombus regression in 62% of patients with LVT treated with OAC.^13^ A further study of 92 LVT patients treated with VKA demonstrated that thrombus resolution was dependent on time spent within the therapeutic range.^14^ However, the narrow therapeutic window of VKAs poses a major problem in therapy necessitating frequent monitoring and dosage adjustments. Treatment with NOACs cannot be recommended at this time due to a lack of robust evidence, despite similar thrombus resolution rates compared to VKAs within our study and in non‐randomized trials.^11^, ^13^, ^14^ Of note, a recently published study by Robinson et al. indicates that anticoagulation with NOACs was associated with a higher risk of ischemic stroke and systemic emboli compared with warfarin treatment in patients with LVT.^15^ However, these results are limited by a lack of randomization and by the retrospective nature of this analysis.^15^

Considering a similar but not identical effect of NOACs and in particular Xa‐inhibitors in resolution of left atrial thrombus (LAT) and LVT resolution, randomized studies assessing LAT resolution provide us some evidence on the potential effect of LVT resolution. In the recently published EMANATE trial, similar LAT resolution rates were reported in patients receiving apixaban (52%) versus patients receiving heparin/VKA (58%).^16^ Nevertheless, a specific effect of NOACs on LVT resolution needs to be proven in future randomized controlled trials (RCT) with a particular focus on the required treatment period.

Surprisingly, 26.7% of patients receiving TAT received ticagrelor/prasugrel as a part of the regimen. Further analysis suggests that TAT with a more potent P2Y_12_ antagonist is associated with a markedly increased likelihood of thrombus resolution. However, a meta‐analysis of several randomized controlled clinical trials (WOEST, PIONEER, RE DUAL, ENTRUST‐AF PCI, and AUGUSTUS) investigating the efficacy and safety of DAT versus TAT in patients with atrial fibrillation undergoing coronary intervention has shown a significantly reduced bleeding risk in patients with DAT compared to TAT and similar efficacy in preventing ischemic events.^17^ In conclusion, TAT with prasugrel/ticagrelor might be an attractive option in patients with persistent LVT after conventional therapy and low‐bleeding risk but future studies are needed to evaluate the bleeding risk in this population.

With respect to clinical endpoints, our data clearly demonstrated a lower risk for MACE, thromboembolic events, and all‐cause death after LVT resolution. Consistent with our findings, Lattuca and colleagues showed that the clinical prognosis of patients with LVT is poor with a very high risk of major cardiovascular events and mortality. Furthermore, they could assess that LVT resolution, obtained with different anticoagulant strategies, was associated with reduced mortality, which may serve as a basis for using LVT resolution as a surrogate endpoint in future RCT.^15^

## 5 LIMITATIONS

Despite the extended follow‐up, several limitations should be addressed.

The major limitations of the present analysis represent its single center setting and the low‐sample size. However, considering the rare occurrence of LVT after MI and long screening period, we obtained a clinically relevant sample size for the present investigation. In addition, patients were not followed using a standardized follow‐up. Also, laboratory measurements were not available for all patients introducing possible selection bias.

Finally, despite multivariable adjustment for clinical variables and biomarkers, residual confounding is possible.

## 6 CONCLUSION

The presented data clearly highlighted the poor outcome of ACS patients experiencing LVT. In terms of personalized risk stratification, the prognostic value of thrombus resolution on MACE, all‐cause death and thromboembolic events can reasonably be considered for risk assessment and treatment decisions in this highly vulnerable patient population. Considering the observation that TAT with more potent P2Y_12_ antagonists was associated with a treatment benefit, an intensified antithrombotic treatment approach might be taken into account in patients with persistent LVT and low‐bleeding risk.

## CONFLICT OF INTEREST

Felix Hofer: none; Niema Kazem: none; Ronny Schweitzer: none; Patricia Horvat: none; Max‐Paul Winter: none; Lorenz Koller: none; Christian; Hengstenberg: none; Patrick Sulzgruber: grants from Daiichi Sankyo and grants from Boehringer‐Ingelheim outside the submitted work; Alexander Niessner: personal fees from Bayer, personal fees from BMS, grants and personal fees from Boehringer Ingelheim, grants and personal fees from Daiichi Sankyo and personal fees from Pfizer outside the submitted work.

## AUTHOR CONTRIBUTIONS

Alexander Niessner, Patrick Sulzgruber, and Felix Hofer contributed to the conception or design of the work. Felix Hofer, Patricia Horvat, Ronny Schweitzer, and Max‐Paul Winter contributed to the acquisition, analysis, or interpretation of data for the work. Felix Hofer drafted the manuscript. Alexander Niessner, Niema Kazem, Lorenz Koller, and Christian Hengstenberg critically revised the manuscript. All gave final approval and agree to be accountable for all aspects of work ensuring integrity and accuracy.

## Data Availability

The data that support the findings of this study are available from the corresponding author upon reasonable request.
